# Translation and Transcultural Adaptation of the Wessex Head Injury Matrix, Italian Version: A Preliminary Report

**DOI:** 10.3390/brainsci11060810

**Published:** 2021-06-18

**Authors:** Francesca Pistoia, Agnes Shiel, Raffaele Ornello, Gennaro Saporito, Luca Gentili, Antonio Carolei, Carmine Marini, Simona Sacco, Franco Marinangeli

**Affiliations:** 1 Neurological Institute, Department of Biotechnology and Applied Clinical Sciences, University of L’Aquila, 67100 L’Aquila, Italy; raffaele.ornello@univaq.it (R.O.); gennaro.saporito@graduate.univaq.it (G.S.); antonio.carolei@univaq.it (A.C.); simona.sacco@univaq.it (S.S.); 2 School of Health Sciences, National University of Ireland, H91 TK33 Galway, Ireland; agnes.shiel@nuigalway.ie; 3 Department of Life, Health and Environmental Science, University of L’Aquila, 67100 L’Aquila, Italy; luca.gentili@graduate.univaq.it (L.G.); carmine.marini@univaq.it (C.M.); franco.marinangeli@univaq.it (F.M.)

**Keywords:** coma, vegetative state, minimally conscious states, consciousness

## Abstract

Background: Patients who are in a coma, a vegetative state or a minimally conscious state present a clinical challenge for neurological assessment, which is a prerequisite for establishing a prognosis and planning management. Several scales have been developed to evaluate these patients. The Wessex Head Injury Matrix is a comprehensive tool but is currently available only in the French and English languages. The aim of this study was to translate and evaluate the reliability of the Italian version of the scale. Methods: The original scale was translated according to a standard protocol: three separate translations were made, and a selected version was back-translated to check for any errors in order to obtain the most accurate Italian translation. A final back translation of the agreed version was made as a further check. The final version was then administered blind to a consecutive series of patients with severe acquired brain injury by two examiners. Inter-rater and test-retest reliability were assessed using a weighted Cohen’s kappa (Kw). Concurrent validity of the WHIM was evaluated by ρ Spearman’s correlation coefficient using the Glasgow Coma Scale (GCS) and the Coma Recovery Scale Revised (CRS-R) as the available gold standard. Results: Twenty-four patients (12 males and 12 females; mean age 59.9 ± 20.1; mean duration from index event 17.7 ± 20.0 days) with stroke (*n* = 15), traumatic brain injury (*n* = 7) and anoxic encephalopathy (*n* = 2) were included. Inter-rater [Kw 0.80 (95% CI 0.75–0.84)] and test-retest reliability [Kw 0.77 (95% CI 0.72–0.81)] showed good values. WHIM total scores correlated significantly with total scores on the GCS (ρ = 0.776; *p* < 0.001) and the CRS-R (ρ = 0.881; *p* < 0.001) demonstrating concurrent validity; Conclusion: The Italian version of the scale is now available for clinical practice and research.

## 1. Introduction

Assessment of patients with disorders of consciousness (coma, unresponsive wakefulness syndrome and minimally conscious state) is challenging due to the complexity of managing patients who survive a severe acquired brain injury. Patients are heterogeneous in relation to age, etiology, brain injury extension and dissemination, and comorbidities [1,2,3]. Moreover, especially in the early phases, they may exhibit a fluctuating behavioral repertoire that makes it difficult to obtain reliable and reproducible assessment results [4]. To further complicate evaluation, patients may be medically unstable and have medical comorbidities including those compromising mobility, such as spasticity, heterotopic ossification, fractures, joint disease and previous disability. These comorbidities, in particular, may hinder purposeful movements and the following of commands that are cardinal signs that can distinguish between unresponsive wakefulness syndrome (UWS) from a minimally conscious state (MCS) [4,5,6,7]. Finally, the presence of other neurological syndromes, such as aphasia, apraxia, hemispatial neglect, paresis and critical illness polyneuropathy or myopathy may prevent the identification of behaviors that indicate the emergence of full consciousness, thus contributing to a high rate of misdiagnoses [8,9]. Further difficulties in the assessment of patients arise from the recent identification of new diagnostic categories based on the use of paraclinical testing (i.e., functional magnetic resonance imaging, positron emission tomography, EEG or evoked potentials), which has led to the identification of a subgroup of patients with a presumed covert awareness who do not show behavioral evidence of consciousness or communication. Specific evidence in this area has been obtained among patients who maintain preserved cognitive skills, such as the ability to perform motor imagery tasks (e.g., tennis playing), despite the absence of any signs of self-expression. To take into account and explain the dissociation between measured bedside behaviors and laboratory investigations, the term cognitive motor dissociation (CMD) has been introduced and a new category of patients has been identified [10,11,12]. This evidence has paved the way for new research directions and, at the same time, confirmed the need for even more extensive assessments of patients, as bedside assessment alone puts clinicians at risk of formulating inaccurate prognoses, increasing the likelihood of withholding life-sustaining therapies or denying rehabilitative services [13].

In order to overcome all the assessment difficulties, many specific behavioral assessment tools have been developed over time: the Glasgow Coma Scale (GCS) allows a rapid assessment of consciousness impairment but does not differentiate between coma and UWS or diagnose MCS reliably; [14] the Rancho Level of Cognitive Functioning Scale (LCFS), has been developed to track recovery and to classify outcome levels; [15] and the Coma Recovery Scale—Revised which is considered the gold standard method for assessing patients with disorders of consciousness—this is the only scale designed specifically to differentiate between UWS and MCS and to capture signs of emergence from UWS to full consciousness (functional communication and functional object use) [16,17,18]. Recently, an additional tool, The Motor Behavior Tool (MBT), has been developed and validated as a complement to the CRS-R to identify subtle motor behavior that is not taken into account in the CRS-R but could be useful in identifying residual cognition in patients: this scale is particularly useful for patients suspected of having CMD because it investigates the degree of residual motor/efference output which potentially interferes with the overall assessment [19,20]. A newer version of the MBT, named MBT-R, with a simplified scoring method, has been introduced and is widely used: its strength is the ability to identify “positive” motor signs as an expression of residual cognition (limb, facial, ocular, or oral intentional or nonreflexive movements appearing spontaneously or in response to the environment or signs of verbal or behavioral interaction, that are not always reproducible) [20].

All the above scales maintain their own peculiarity and utility across the spectrum of disorders of consciousness and along the clinical continuum of recovery.

The Wessex Head Injury Matrix (WHIM), although different from previous tools because it was not developed for diagnostic purposes, has been found to be useful in clinical practice and research [21]. Its strength is that it is designed to collect data by observation and by testing skills used in daily life. As previously highlighted, it is not a diagnostic tool, so it cannot be used in place of other scales, especially the CRS-R and the MBT-r, but it can be of help as a behavioral assessment schedule to assess and monitor recovery in patients. In the past, the WHIM has been used to characterize patients both in neurophysiological studies searching for the electrophysiological correlates of consciousness in severe acute brain damage and in some studies evaluating outcomes [22,23]. Its use has been considered noteworthy also in research protocols investigating the effects of sensory stimulation evoked by the use of biographically meaningful objects and the effects of intensive physiotherapy on the long-term well-being of patients with an acquired brain injury [24,25]. Other studies have suggested the usefulness of serial WHIM assessments to identify the trajectory of changes that is most likely to predict the final outcome and to investigate how physical changes, such as infections and comorbidities, can affect recover [26,27].

Currently, the WHIM is available only in English and French [21,22,23,24,25,26,27,28].

The objective of this study was to develop and evaluate the reliability of the Italian version of the WHIM and to make it available for clinical practice and research.

## 2. Materials and Methods

The following standard protocol [29] was used to develop an Italian version of the WHIM:

(a) Preparation of the work and invitation of the instrument developer to be involved.
(b) Forward translation: three authors from the research group completed three separate translations of the original scale.
(c) Reconciliation: comparison and merging of forward translations into a single forward translation.
(d) Back translation of the selected version to check for any errors which may have occurred during the original translation.
(e) Comparison of the bask-translated version with the original one to investigate any discrepancies
(f) Harmonization to resolve any discrepancies
(g) Consensus meeting, with particular attention being given to the level of consistency of the new scale with the original one, to highlight and amend discrepancies.
(h) Proofreading and finale report.

For the reliability study, all patients consecutively admitted to the neurological and stroke unit and the intensive care unit of the San Salvatore Hospital of L’Aquila within a one-year period were screened.

Inclusion criteria were: age >18 years; diagnosis of a disorder of consciousness as a result of a severe brain injury, defined as a score of 8 or less on the GCS in the acute phase; no past medical history of neurological or psychiatric diseases or learning disabilities; signed informed consent provided by the legal representative of the patient. Patients who were medically unstable due to acute complications and those taking drugs interfering with arousal and consciousness were excluded. The final sample comprised 24 patients, all fulfilling the inclusion criteria.

Diagnostic reliability between raters (inter-rater reliability) and ratings (intrarater reliability) was investigated as follows: the final Italian version of the scale was administered to all the included patients by two examiners on the same day. The two raters assessed each patient independently and recorded single-item subscores. Each rater was blind to the information collected by the other. After 2 days, a second evaluation by the first rater was made in order to estimate test-retest reliability. The two raters were blinded to the diagnosis of the state of consciousness, that was made by a third examiner using the GCS and the CRS-R. The concurrent validity of the WHIM was evaluated by ρ Spearman’s correlation coefficient using the GCS and the CRS-R as the available gold standard. Pertinent demographic and clinical data were also collected.

The research protocol was approved by the Internal Review Board of the University of L’Aquila (n. 52/2019).

### Statistical Analysis

Descriptive statistics are reported as mean ± standard deviation (SD) or median with interquartile range (IQR) for quantitative variables and as counts and proportions (%) for categorical variables. Inter-rater and test-retest reliability were assessed using the weighted Cohen’s kappa (K). k-values were interpreted as it follows: >0.80 excellent agreement, 0.61–0.80 good agreement, 0.41–0.60 moderate agreement, 0.21–0.40 fair agreement, and <0.21 poor agreement. Concurrent validity was tested using a Spearman rank order correlation between the WHIM scores and the GCS and CRS-R scores. The Spearman correlation coefficient (rs) can take values from +1 to −1: a coefficient of +1 indicates a perfect association of ranks, a coefficient of zero indicates no association between ranks and a coefficient of −1 indicates a perfect negative association of ranks. The closer coefficient is to zero, the weaker the association between the ranks. Data analysis was performed using the IBM SPSS Statistics 20.0. The level of significance was fixed at 0.05.

## 3. Results

The final version of the WHIM is reported in Table 1.

For the reliability study, twenty-four patients (12 males and 12 females; mean age 59.9 ± 20.1; mean duration from index event 17.7 ± 20.0 days) were investigated. Consciousness impairment was caused in most cases by stroke (*n* = 15), followed by traumatic brain injury (*n* = 7) and anoxic encephalopathy (*n* = 2). Anagraphical and clinical characteristics of patients are reported in Table 2.

The mean inter-rater [Kw 0.80 (95% CI 0.75–0.84)] and test-retest reliability [Kw 0.77 (95% CI 0.72–0.81)] showed good values. Kappa coefficients could not be computed for twelve items as there was no variability in the assessments. Inter-rater agreement was excellent for 38% of the remaining items (K = 0.8–1), moderate to good for 46% (K = 0.41–0.80) and poor to fair for 16% (K <0.40). Test-retest agreement was excellent for 50% of the items (K = 0.8–1), fair to good for 44% (K = 0.41–0.80) and poor for 6% (K <0.40). Distribution of Kappa coefficients for the single items are shown in Table 3.

WHIM total scores correlated significantly with total scores on the GCS (ρ = 0.776; *p* < 0.001) and the CRS-R (ρ = 0.881; *p* < 0.001) demonstrating concurrent validity.

## 4. Discussion

The Italian version of the WHIM has been shown to be an additional useful instrument to characterize patients with disorders of consciousness in Italy. Although it cannot be considered as a diagnostic tool, it can be added to the comprehensive battery of scales that is available for the evaluation of these challenging patients. The WHIM has several strengths, not least that it is essentially based on observation of the patient without necessarily resorting to external stimuli [21]. This allows examiners to overcome difficulties arising from the possible presence of coexisting neurological symptoms such as spasticity, aphasia and motor impairments that may prevent accurate assessment of consciousness [2,9]. Moreover, the WHIM can be used to explore the patient’s behavior from various perspectives and using a variety of modalities such as auditory, visual, verbal and motor, thus allowing for the difficulties arising when one or more of these modalities is not viable [21]. This scale divides the time of recovery into small steps, and items are fine-grained enough to capture even small improvements that can be used in the progressive update of rehabilitation goals [21]. Moreover, the WHIM can detect not only improvements but also deterioration, when a loss of previously observed behaviors occurs. This is because behavior is merely observed and scheduled and is evaluated as such without making decisions about its purposeful nature. Therefore, the scale looks like a behavioral assessment schedule, which helps in identifying the more likely sequence of recovery, usually starting with an increase in arousal and alerting followed first by an enhancement of selective attention and subsequently by the reappearance of divergent thinking and reasoning [21].

Due to the complexity of patients with disorders of consciousness following a severe acquired brain injury, using multiple assessment tools may be of help in detecting consciousness transitions. The gold standard tool to investigate patients with UWS or MCS remains the CRS-R, which is a unique scale that can capture signs of emergence from MCS to full consciousness. Therefore, the aim of this study was not to provide an alternative to the CRS-R but to make available an additional tool for clinical practice and research, through the process of translation and cross-cultural adaptation of the original version of the WHIM. Indeed, this scale has not previously been proposed as a diagnostic tool but it has been used as an instrument to collect data obtained by observation. Therefore, it does not represent an alternative to other diagnostic tools, but it may contribute to enriching the assessment options available. It may also represent a useful implement enabling a more careful evaluation of cases that are susceptible to misdiagnosis or uncertain prognosis. Based on the strengths and the limitations of the WHIM, it would be interesting to propose and test a model of a symbiotic relationship between the WHIM and the other scales, such as the CRS-R and the MBT-r, that have been specifically developed for diagnostic purposes; this might increase our ability to detect even subtle signs of consciousness recovery that may be missed with the isolated use of single scales, especially when cognitive motor dissociation is suspected [2,9,12].

Strengths of our study include the application of a rigorous standard protocol for translation and cross-cultural adaptation of the scale. A comprehensive multistep process lead to achieve cross-cultural equivalence between the instrument in the source language and the instrument in the target language. Any ambiguities and discrepancies were discussed and resolved using a committee approach to assure conceptual, semantic and content equivalence of the translated instrument as compared to the original one. This contributed to the process of harmonization of the final version.

Possible limitations of this study include the choice of a subacute setting to perform reliability analysis and the absence of a full psychometric testing of the translated instrument. We selected this setting because the main difficulty in assessing severely injured patients is just in the weeks immediately after the injury, when small gains may go unnoticed. On the other hand, as patients progress along the path of consciousness recovery may exhibit behaviors belonging to the sphere of activities of daily living that are more easily identifiable. However, the choice of a subacute setting for reliability analysis may represent a confounding factor, especially when analyzing the test-retest reliability using a two-day interval between the two tests. As patients can significantly change in the acute phase of injury, we paid particular attention to this issue, and so excluded from the study all patients who were medically unstable due to acute complications and those taking drugs interfering with arousal and consciousness. Furthermore, we monitored any changes in diagnosis (UWS, MCS, full consciousness) using the GCS and the CRS-R. This approach allowed us to exclude changes that could have interfered with test-retest reliability. An interval of less than 48 h to estimate test-retest reliability could have affected the final analysis due to the effect of memory in recalling previous answers. A possible further limitation lies with the missed use of the MBTr, as a complement tool to the CRS-R, to make diagnosis in the acute phase: this might have contributed to properly assess signs of conscious perception especially in patients with possible CMD. Finally, another limitation lies with the smaller sample of patients used to investigate inter-rater and test-retest reliability. However, the aim of the preliminary study was not the validation of the scale: this will be the object of future research protocols investigating the complete psychometric properties of the scale in a large sample of patients across the complete spectrum of disorders of consciousness (coma, UWS and MCS) both in acute and subacute settings.

Therefore, our findings pave the way for future studies exploring the usefulness of the Italian version of the scale along the whole pathway of recovery of disorders of consciousness. The availability of an additional instrument, not to be used as a substitute for the other diagnostic tools, may increase our chances of formulating a prognosis reliably and capturing even subtle signs of consciousness transitions, that may indicate either improvement or possible deterioration. Using the scale, it could be interesting to explore which behavioral sequence is more likely to lead to a relevant degree of recovery at the end of the assessment period and what are the intervals between the onset of the various observed behaviors. Further studies are necessary in order to endorse our findings and to investigate the full psychometric properties of the scale.

## Figures and Tables

**Table 1:** Wessex Head Injury Matrix (62 Items), Italian Version.

**Nome e Cognome:**		**____________________**		
**Data di nascita:**		**____________________**		
**Ospedale:**		**____________________**		
**Reparto:**		**____________________**		
Iniziare dall’*item* numero 1. Fare un segno di spunta in corrispondenza di tutti i comportamenti osservati ed inserire una croce per quelli non osservati. Una volta raggiunte 10 croci consecutive, interrompere la valutazione. Registrare come punteggio finale il numero del comportamento più evoluto che è stato osservato.				
			**DATA**	
	Punteggio WHIM			N. del comportamento più evoluto.
N.	COMPORTAMENTI OSSERVATI			DEFINIZIONI OPERATIVE
1	Breve apertura degli occhi.			Meno di 30 s.
2	Apertura sostenuta degli occhi.			Più di 30 s.
3	Gli occhi sono aperti e si muovono ma non fissano una persona o un oggetto.			Gli occhi si muovono in maniera aleatoria. Non ci sono segni di inseguimento visivo e gli occhi non si fermano a fissare oggetti o persone.
4	Attenzione catturata momentaneamente da uno stimolo predominante.			Stimolo saliente presente per due secondi o più = rumoroso/di significative dimensioni/dai colori vivaci/doloroso. Cambiamento identificabile nel comportamento, sebbene momentaneo, come passaggio da stato di agitazione a stato di calma, apertura degli occhi, passaggio da immobilità a comparsa di comportamenti motori.
5	Il paziente guarda brevemente una persona.			Guarda = gli occhi si muovono in modo causale nella stanza… quando un oggetto o una persona viene notato gli occhi si fermano su esso. Brevemente, per qualche istante, si ha l’impressione che guardi verso qualcosa o qualcuno.
6	Vocalizzazione volontaria per esprimere degli stati d’animo.			Gemiti o lamenti come per esprimere un malessere, sia spontanei sia evocati da procedure messe in atto in quel momento come mobilizzazione passiva di arti contratti, iniezioni o prelievi di sangue.
7	Digrignare o serrare i denti.			Digrigna i denti spontaneamente o quando è inserito in bocca un tampone. I denti si serrano in risposta ad un tampone in schiuma inserito in bocca.
8	Avere un contatto visivo.			Il paziente reagisce al suono del suo nome pronunciato da una persona al di fuori del suo campo visivo, dirigendo il suo sguardo su questa persona e mantenendolo per almeno 3 s.
9	Il paziente guarda la persona che sta parlando con lui.			Sposta lo sguardo sulla persona che gli sta parlando. Continua a guardarla per almeno 3 s.
10	Esclamazione di parolacce (“va a quel paese”, etc).			“Va a quel paese”, etc.
11	Stato di allerta e agitazione prima della minzione o della defecazione.			Il paziente diventa estremamente irrequieto e agitato prima di urinare o defecare. Il paziente si calma subito dopo.
12	Gli occhi seguono una persona che si muove nel suo campo visivo.			Gli occhi del paziente seguono una persona che si muove dal centro del suo campo visivo verso destra o verso sinistra. L’inseguimento visivo non deve per forza spaziare nell’intero campo visivo.
13	Guarda la persona che si prende cura di lui.			Il suo sguardo si sofferma per almeno tre secondi sulla persona che si sta prendendo cura di lui (ad esempio nell’atto di sistemare il letto o di mobilizzare gli arti).
14	Vocalizzi riflessi (durante un sospiro o uno sbadiglio, etc).			Deve essere prodotto un suono, per cui, uno sbadiglio silenzioso non può essere considerato. Il paziente deve essere in grado di produrre un suono normale quando tossisce.
15	Eseguire un movimento su richiesta verbale.			Obbedisce a un comando su richiesta verbale: comando singolo (esempio: alza il braccio).
16	Gira la testa o gli occhi per guardare qualcuno che parla.			Inizialmente lo sguardo del paziente è diretto altrove. Muove gli occhi o gira la testa per guardare qualcuno che parla. La persona che parla non deve farlo necessariamente con il paziente.
17	Guarda una persona che si muove nel suo campo visivo.			La persona si muove da un lato del letto all’altro passando al davanti del letto. Gli occhi del paziente seguono dal fondo del letto verso destra o verso sinistra o in entrambe le direzioni.
18	Aggancia qualcosa con lo sguardo per 3–5 secondi.			Attrarre l’attenzione del paziente con un oggetto grande dai colori vivaci e muoverlo attraverso il campo visivo. Segnalare il comportamento se il paziente segue per almeno 90°.
19	Parla sussurrando.			Il paziente si esprime sussurrando.
20	Vocalizza per esprimere i suoi bisogni o stati d’animo.			Vocalizza come per comunicare bisogni o stati d’animo, spontaneamente o in modo evocato da procedure fastidiose come prelievi del sangue, iniezioni, ginnastica posturale ecc.
21	Pianto.			Il paziente piange, le lacrime possono essere presenti o assenti.
22	Individua una fonte sonora.			Suono di un campanello, di un fischietto, di un cercapersone o di un oggetto simile. Il paziente gira la testa o gli occhi verso la fonte sonora.
23	Mostra risposte specifiche nei confronti di particolari persone.			Nei confronti dei familiari quando un esaminatore è presente… obbedisce ai comandi su richiesta dei familiari, collabora con loro ma non con lo staff sanitario, si tranquillizza in presenza di un familiare o, al contrario, diventa più rumoroso in loro presenza. Si mostra più rilassato e collaborante con alcuni membri dello staff sanitario piuttosto che con altri.
24	Mantiene il contatto visivo per cinque secondi.			Guarda una persona per cinque secondi o più a lungo.
25	Riproduce parole con le labbra senza emettere suoni.			Mima le parole con la bocca (esempio ciao). Questo comportamento non contempla i movimenti di masticazione.
26	Corruccia il volto, fa smorfie ecc per mostrare disappunto.			Si osserva quando il paziente è sottoposto ad un prelievo di sangue, a fisioterapia respiratoria (specialmente durante le manovre di aspirazione), a mobilizzazione passiva se ha aumento del tono e contratture, se messo seduto o posto sul lettino verticalizzabile.
27	E’ capace di ignorare fonti di distrazione.			Quando il paziente sta prestando attenzione, per esempio nel guardare qualcuno che gli parla, è in grado di ignorare adeguatamente fonti di distrazioni, per esempio quelle rappresentate da persone che entrano nella stanza.
28	Guarda un oggetto se richiesto.			Porre un oggetto vivacemente colorato in un punto che il paziente non può vedere e chiedergli di guardarlo.
29	Sceglie un oggetto se richiesto.			Scegliere due oggetti, entrambi situati nel campo visivo del paziente ma sufficientemente lontani tra loro in modo che debba muovere gli occhi nel passaggio dall’uno all’altro. Chiedere al paziente di guardare l’oggetto a destra. Chiedere al paziente di guardare l’oggetto a sinistra. Invertire gli oggetti e far ripetere il compito.
30	Ride.			Il paziente produce dei suoni o dei movimenti per esprimere il suo divertimento, può apparire appropriato o inappropriato.
31	Imita gesti (ammiccamento palpebrale, tiene il pollice rivolto verso l’alto etc).			Chiedere al paziente di imitare i gesti eseguiti dall’esaminatore, accompagnati da istruzioni verbali. Deve essere chiaro che il paziente lo fa su richiesta (ripetere la procedura per confermare se si è in dubbio).
32	Indica di aver capito scrollando la testa, facendo cenni con il capo o altri gesti.			Presentando una lista di 10 domande, stabilire se il paziente può rispondere in modo affidabile riproducendo un si o un no. Dovrà fornire 9 risposte corrette su 10.
33	Cerca il contatto visivo.			Muove la testa e gli occhi nel tentativo di trovare un contatto visivo e lo mantiene per almeno tre secondi.
34	Usa monosillabi o parole singole in risposta a domande.			Singole sillabe o singole parole…. Si, no, etc…
35	Guarda, apparentemente esplorandole con lo sguardo, foto, giornali, TV etc.			Uso di immagini: ad esempio, foto di famiglia. Ne guarda una, la posa, ne guarda un’altra. Uso del giornale: gira le pagine. TV: guarda per una durata di tempo appropriata la scena di un programma, la durata di una pubblicità o di un clip video.
36	Sposta lo sguardo da una persona all’altra spontaneamente.			Due persone sono nella camera in modo tale che il paziente debba muovere gli occhi o la testa per volgere lo sguardo dall’una all’altra. Passa spontaneamente dall’una all’altra con lo sguardo.
37	Il linguaggio è fluente ma sconclusionato. Usa molte parole ma il significato globale è difficilmente comprensibile.			Il paziente passa da un argomento ad un altro o fornisce dettagli superflui, non risponde alle domande o non si attiene al tema della conversazione.
38	Cerca oggetti che vengono dapprima mostrati e poi rimossi dal campo visivo.			Al paziente viene presentato, per almeno 15 secondi, un oggetto grande, molto luminoso e per lui significativo; successivamente, l’oggetto viene rimosso e nascosto (ad esempio: sotto le lenzuola del letto, comunque a portata di mano del paziente). Il paziente può utilizzare qualsiasi mezzo per indicare dove si trova l’oggetto (gesti, parole, movimenti oculari, movimenti per indicare).
39	Può partecipare ad alcune attività (per esempio guardare la TV ecc) ma la concentrazione è labile. Ogni stimolo esterno può essere distraente.			Partecipa ad un compito per un minuto senza distrazioni. Qualsiasi distrattore inficia immediatamente la sua attenzione, senza che torni al compito originale.
40	Usa monosillabi o parole per esprimere bisogni o stati d’animo.			“Stanco”, “Fame”, “Sete”, “Dolore” etc...le parole sono usate singolarmente senza costruzione di frasi.
41	Viene momentaneamente distratto da stimoli esterni ma può riprendere il compito.			Momentaneamente = non più di 10 secondi.
42	Può identificare una certa carta da gioco su una selezione di quattro carte.			Vengono presentate 4 carte da gioco: 2 nere, 2 rosse ciascun paio raffigurante numeri o figure. Chiedere al paziente di selezionarne una. 10 tentativi.
43	Sorrisi.			Sorride spontaneamente, non importa per quale ragione.
44	Usa la scrittura, una tastiera o un altro mezzo di comunicazione, ma è difficile da comprendere.			Prova a scrivere il suo nome o una parola. Scrive anziché firmare. Alcune lettere sono riconoscibili.
45	Può indicare il momento della giornata.			Dare al paziente tre opzioni: mattino, pomeriggio, sera, oppure utilizzare i pasti come marcatori temporali (colazione, pranzo, cena). Esempio: dopo colazione, prima del pranzo ecc…
46	Brevi sequenze di parole.			Frasi—no proposizioni complete o comunque frasi confuse.
47	Indica con gli occhi.			Scegliere tra due immagini o due oggetti o due carte (SI/NO). Gli occhi devono indicare correttamente in 9 prove su 10.
48	Avviare la conversazione.			Attrae l’attenzione di un’altra persona emettendo suoni o facendo gesti (non è necessario un linguaggio vero e proprio).
49	Vocalizza per attirare l’attenzione.			In una situazione in cui le persone nella stanza non prestano attenzione al paziente, per esempio perché stanno parlando tra loro, il paziente produce un suono, non importa quale, per attirare l’attenzione.
50	Il linguaggio è preservato ma i contenuti riflettono problemi nel reperimento di vocaboli o nella comprensione.			Il paziente può eseguire due ordini semplici alla volta, ma non di più. (Es: Alza il braccio e chiudi la mano). Ha difficoltà nell’esprimersi e nel denominare gli oggetti.
51	Usa un linguaggio convenzionale ma con poche parole.			Frasi abbreviate. Indica solo i fatti, dando poca o nessuna descrizione.
52	Usa uno o due gesti.			Pollice all’insù/giù Cenno con il capo/scrollare il capo Può essere un comportamento spontaneo o in risposta ad una sollecitazione.
53	Fornisce, in maniera corretta, uno o due indicatori d’orientamento (giorno, mese, anno, età, luogo).			Chiedere al paziente: In quale giorno ci troviamo? in quale mese ci troviamo? Quanti anni hai? Dove ti trovi adesso (ospedale) e in che città? Se il paziente non sa rispondere, fornire le indicazioni corrette.
54	Conosce il prezzo di tre articoli comuni (pane, latte, birra) di poco prezzo.			Oggetti: CD audio, barrette di Mars, lattine di Coca-Cola. Il prezzo corretto deve essere espresso per i tre oggetti nello stesso momento.
55	Riconosce le monete indicandole tramite movimenti oculari o con il tocco.			Vengono mostrate tre monete: 5 centesimi, 50 centesimi, 1 euro poste su una riga e presentate nel seguente ordine: 50 centesimi, 5 centesimi, 1 euro: il paziente deve indicare le monete in questo ordine: 5 centesimi, 1 euro, 50 centesimi.
56	Conosce il nome di un membro del team.			Chiama spontaneamente e per nome un membro dell’équipe medica o ricorda il suo nome quando gli viene chiesto. Verificare che il paziente non stia leggendo il nome sul badge.
57	Denomina e indica la sua destra e la sua sinistra.			Esegue le istruzioni (es: “Alza la tua mano sinistra”, “ Alza la tua mano destra”, “ Gira la testa a destra”, “Gira la testa a sinistra” o comandi simili).
58	Usa la scrittura, una tastiera o un altro mezzo di comunicazione in maniera fluente.			I suoi messaggi sono facilmente comprensibili da chiunque.
59	Fornisce da 3 a 5 indicatori di orientamento corretti.			Procedura e indicatori di orientamento descritti sopra.
60	Ricorda qualcosa del giorno precedente. (esempio: Mostrare al paziente una moneta, una chiave, un orologio e dirgli che gli verrà chiesto di ricordarlo il giorno dopo).			Mostrare l’oggetto al paziente, metterlo in tasca e dire al paziente che il giorno dopo gli sarà chiesto cosa c’è nella tasca. Il giorno successivo chiedere al paziente se ricorda l’oggetto. Se il paziente non ricorda, dare tre opzioni “era un orologio, una chiave o una moneta?”. Segnalare il comportamento se il paziente risponde bene.
61	Ricorda qualcosa avvenuto nella parte precedente della giornata (esempio: sei stato a fare fisioterapia?).			Verificare cosa ha fatto il paziente durante la giornata. Scegliere un evento che non si verifichi indifferentemente di mattina o di pomeriggio. Vengono fornite domande aperte come: “Che cosa hai fatto questa mattina?”. Se non si riceve risposta, chiedere dando uno spunto ulteriore come “Che cosa hai fatto durante la fisioterapia questa mattina?”.
62	Completare test per amnesia post-traumatica.			Il paziente è uscito dalla fase di amnesia post-traumatica.

For publication of the scale the rights in the material are owned by SAGE and permission for any further reuse must be obtained from the relevant holder of the exclusive rights.

**Table 2:** Anagraphical and clinical characteristics of included patients for reliability analysis (ICH: Intracerebral Hemorrhage, TBI: Traumatic Brain Injury, PAE: Post-anoxic encephalopathy, IS: Ischemic Stroke: SAK: Subarachnoid Hemorrhage, UWS: Unresponsive Wakefulness Syndrome, MCS: Minimally Conscious State). The diagnosis of the state of consciousness was made using the Glasgow Coma Scale (GCS) and the Coma Recovery Scale—Revised (CRS-R).

N.	Sex	Age Range (Years)	Primitive Injury	Time from Injury to Evaluation (Days)	Diagnosis	GCS—CRS-R (Test Evaluation)	GCS—CRS-R (Re-Test Evaluation)
1	Male	65–79	ICH	5	Coma	3 (GCS)—0 (CRS-R)	3 (GCS)—0 (CRS-R)
2	Male	50–64	TBI	39	MCS	11 (GCS)—9 (CRS-R)	11 (GCS)—8 (CRS-R)
3	Female	65–79	PAE	30	Coma	3 (GCS)—0 (CRS-R)	3 (GCS)—0 (CRS-R)
4	Male	65–79	TBI	51	UWS	10 (GCS)—12 (CRS-R)	11 (GCS)—12 (CRS-R)
5	Male	35–49	TBI	10	UWS	8 (GCS)—8 (CRS-R)	8 (GCS)—8 (CRS-R)
6	Female	≥80	TBI	9	Coma	4 (GCS)—5 (CRS-R)	4 (GCS)—5 (CRS-R)
7	Male	65–79	SAH	2	MCS	6 (GCS)—5 (CRS-R)	6 (GCS)—5 (CRS-R)
8	Male	65–79	SAH	13	UWS	4 (GCS)—5 (CRS-R)	4 (GCS)—5 (CRS-R)
9	Female	≥80	IS	5	Coma	5 (GCS)—6 (CRS-R)	5 (GCS)—6 (CRS-R)
10	Male	35–49	SAH	6	MCS	13 (GCS)—19 (CRS-R)	13 (GCS)—18 (CRS-R)
11	Female	≥80	SAH	2	Coma	0 (GCS)—0 (CRS-R)	0 (GCS)—0 (CRS-R)
12	Male	18–24	TBI	11	Coma	5 (GCS)—6 (CRS-R)	5 (GCS)—6 (CRS-R)
13	Female	50–64	SAH	20	UWS	14 (GCS)—17 (CRS-R)	14 (GCS)—17 (CRS-R)
14	Female	65–79	ICH	22	Coma	6 (GCS)—6 (CRS-R)	6 (GCS)—6 (CRS-R)
15	Male	35–49	ICH	20	MCS	14 (GCS)—17 (CRS-R)	14 (GCS)—17 (CRS-R)
16	Male	35–49	ICH	7	Coma	4 (GCS)—2 (CRS-R)	4 (GCS)—3 (CRS-R)
17	Male	50–64	SAH	16	Coma	3 (GCS)—0 (CRS-R)	3 (GCS)—0 (CRS-R)
18	Male	25–34	SAH	9	MCS	8 (GCS)—14 (CRS-R)	8 (GCS)—14 (CRS-R)
19	Male	65–79	SAH	5	Coma	10 (GCS)—5 (CRS-R)	10 (GCS)—5 (CRS-R)
20	Female	65–79	TBI	9	MCS	11 (GCS)—15 (CRS-R)	11 (GCS)—15 (CRS-R)
21	Female	50–64	SAH	12	Coma	5 (GCS)—6 (CRS-R)	5 (GCS)—6 (CRS-R)
22	Female	≥80	TBI	14	Coma	5 (GCS)—5 (CRS-R)	5 (GCS)—5 (CRS-R)
23	Female	≥80	SAH	15	UWS	7 (GCS)—6 (CRS-R)	7 (GCS)—6 (CRS-R)
24	Female	25–34	PAE	94	UWS	8 (GCS)—6 (CRS-R)	8 (GCS)—6 (CRS-R)

**Table 3:** Distribution of Kappa coefficients for the single items of the scale (items with absence of variability are not reported in the table).

	Inter-Rater Reliability	Test-Retest Reliability
K > 80 excellent agreement	Items n. 4, 5, 6, 8, 12, 13, 17, 22, 27, 31, 32, 33, 35, 36, 37, 39, 49, 50, 51	Items n. 1, 2, 6, 8, 9, 10, 13, 17, 25, 27, 30, 32, 35, 37, 38, 40, 43, 44, 48, 49, 50, 51, 52, 57, 62
0.61 < k < 0.80 good agreement	Items n. 1, 2, 9, 15, 16, 18, 20, 24, 25, 28, 29, 30, 38, 40, 44, 46, 47, 48, 52, 62	Items n. 3, 4, 5, 7, 12, 14, 16, 18, 19, 22, 24, 28, 29, 31, 39
0.41 < k < 0.60 moderate agreement	Items n. 14, 43, 57	Items n. 15, 33, 34, 36, 46, 47, 45
0.21 < k < 0.40 fair agreement	Items n. 3, 7, 10, 19, 26, 34	Items n. 20, 26
K < 0.21 poor agreement	Items n. 23, 45	Items n. 23

## Data Availability

Data are available on request to the corresponding author.

## References

[1] Scarponi F., Zampolini M., Zucchella C., Bargellesi S., Fassio C., Pistoia F., Bartolo M. (2019). Identifying clinical complexity in patients affected by severe acquired brain injury in neurorehabilitation: A cross sectional survey. Eur. J. Phys. Rehabil. Med..

[2] Sarà M., Pistoia F. (2010). Defining consciousness: Lessons from patients and modern techniques. J. Neurotrauma.

[3] Pistoia F., Sacco S., Franceschini M., Sarà M., Pistarini C., Cazzulani B., Simonelli I., Pasqualetti P., Carolei A. (2015). Comorbidities: A key issue in patients with disorders of consciousness. J. Neurotrauma.

[4] Giacino J.T., Whyte J. (2005). The vegetative state and minimally conscious state: Current knowledge and remaining questions. J. Head Trauma Rehabil..

[5] Estraneo A., Masotta O., Bartolo M., Pistoia F., Perin C., Marino S., Lucca L., Pingue V., Casanova E., Romoli A. (2021). Multi-center study on overall clinical complexity of patients with prolonged disorders of consciousness of different etiologies. Brain Inj..

[6] Pistoia F., Carolei A., Bodien Y.G., Greenfield S., Kaplan S., Sacco S., Pistarini C., Casalena A., De Tanti A., Cazzulani B. (2019). The Comorbidities Coma Scale (CoCoS): Psychometric Properties and Clinical Usefulness in Patients with Disorders of Consciousness. Front. Neurol..

[7] Estraneo A., Pascarella A., Masotta O., Bartolo M., Pistoia F., Perin C., Marino S., Lucca L., Pingue V., Casanova E. (2021). Multi-center observational study on occurrence and related clinical factors of neurogenic heterotopic ossification in patients with disorders of consciousness. Brain Inj..

[8] Giacino J.T., Schnakers C., Rodriguez-Moreno D., Kalmar K., Schiff N., Hirsch J. (2009). Behavioral assessment in patients with disorders of consciousness: Gold standard or fool’s gold?. Prog. Brain Res..

[9] Pistoia F., Sarà M., Saco S., Carolei A. (2013). Vegetative states and minimally conscious states revisited: A Russian doll approach. Brain Inj..

[10] Bruno M.A., Vanhaudenhuyse A., Thibaut A., Moonen G., Laureys S. (2011). From unresponsive wakefulness to minimally conscious PLUS and functional locked-in syndromes: Recent advances in our understanding of disorders of consciousness. J. Neurol..

[11] Schnakers C., Hirsch M., Noé E., Llorens R., Lejeune N., Veeramuthu V., De Marco S., Demertzi A., Duclos C., Morrissey A.M. (2020). Covert Cognition in Disorders of Consciousness: A Meta-Analysis. Brain Sci..

[12] Schiff N.D. (2015). Cognitive Motor Dissociation Following Severe Brain Injuries. JAMA Neurol..

[13] Edlow B.L., Chatelle C., Spencer C.A., Chu C.J., Bodien Y.G., O’Connor K.L., Hirschberg R.E., Hochberg L.R., Giacino J.T., Rosenthal E.S. (2017). Early detection of consciousness in patients with acute severe traumatic brain injury. Brain.

[14] Teasdale G., Jennett B. (1974). Assessment of coma and impaired consciousness: A practical scale. Lancet.

[15] Gouvier W.D., Blanton P.D., LaPorte K.K., Nepomuceno C. (1987). Reliability and validity of the dis-ability rating scale and the levels of cognitive functioning scale in monitoring recovery from severe head injury. Arch. Phys. Med. Rehabil..

[16] Giacino J.T., Kalmar K., Whyte J. (2004). The JFK Coma Recovery Scale-Revised: Measurement Characteristics and Diagnostic Utility. Arch. Phys. Med. Rehabil..

[17] Lombardi F., Gatta G., Sacco S., Muratori A., Carolei A. (2007). The Italian version of the Coma Recovery Scale-Revised (CRS-R). Funct. Neurol..

[18] Sacco S., Altobelli E., Pistarini C., Cerone D., Cazzulani B., Carolei A. (2011). Validation of the Italian version of the Coma Recovery Scale-Revised (CRS-R). Brain Inj..

[19] Pignat J.M., Mauron E., Johr J., Gilart de Keranflec’h C., Van De Ville D., Preti M.G., Meskaldji D.E., Hömberg V., Laureys S., Draganski B. (2016). Outcome prediction of consciousness disorders in the acute stage based on a complementary motor behavioural tool. PLoS ONE.

[20] Pincherle A., Jöhr J., Chatelle C., Pignat J.M., Du Pasquier R., Ryvlin P., Oddo M., Diserens K. (2019). Motor behavior unmasks residual cognition in disorders of consciousness. Ann. Neurol..

[21] Shiel A., Horn S.A., Wilson B.A., Watson M.J., Campbell M.J., Mclellan D.L. (2000). The Wessex Head Injury Matrix (WHIM) main scale: A preliminary report on a scale to assess and monitor patient recovery after severe head injury. Clin. Rehabil..

[22] Schnakers C., Majerus S., Laureys S. (2005). Bispectral analysis of electroencephalogram signals during recovery from coma: Preliminary findings. Neuropsychol. Rehabil..

[23] Wilson F.C., Elder V., McCrudden E., Caldwell S. (2009). Analysis of Wessex Head Injury Matrix (WHIM) scores in consecutive vegetative and minimally conscious state patients. Neuropsychol. Rehabil..

[24] Di Stefano C., Cortesi A., Masotti S., Simoncini L., Piperno R. (2012). Increased behavioural responsiveness with complex stimulation in VS and MCS: Preliminary results. Brain Inj..

[25] Wheatley-Smith L., McGuinness S., Colin Wilson F., Scott G., McCann J., Caldwell S. (2013). Intensive physiotherapy for vegetative and minimally conscious state patients: A retrospective audit and analysis of therapy intervention. Disabil. Rehabil..

[26] Turner-Stokes L., Bassett P., Rose H., Ashford S., Thu A. (2015). Serial measurement of Wessex Head Injury Matrix in the diagnosis of patients in vegetative and minimally conscious states: A cohort analysis. BMJ Open.

[27] Dhamapurkar S.K., Wilson B.A., Rose A., Florschutz G., Watson P., Shiel A. (2018). Does a regular Wessex Head Injury Matrix assessment identify early signs of infections in people with Prolonged Disorders of Consciousness?. Brain Inj..

[28] Majerus S., Vanr De Linden M., Shiel A. (2000). Wessex head Injury Matrix and Glasgow/Glasgo- Liege Coma scale. A validation and comparison study. Neuropsychol. Rehabil..

[29] Wild D., Grove A., Martin M., Eremenco S., McElroy S., Verjee-Lorenz A., Erikson P., ISPOR Task Force for Translation and Cultural Adaptation (2005). Principles of Good Practice for the Translation and Cultural Adaptation Process for Patient-Reported Outcomes (PRO) Measures: Report of the ISPOR Task Force for Translation and Cultural Adaptation. Value Health.

